# Optical digital twins for disease prevention, diagnosis, therapy, and intervention

**DOI:** 10.1117/1.JBO.31.8.080601

**Published:** 2026-08-05

**Authors:** Aydogan Ozcan, Melissa C. Skala, Brian W. Pogue, Jürgen Popp, Laura Marcu, Colleen E. Clancy

**Affiliations:** a University of California Los Angeles, Electrical & Computer Engineering Department, Los Angeles, California, United States; b Morgridge Institute for Research, Madison, Wisconsin, United States; c University of Wisconsin, Department of Biomedical Engineering, Madison, Wisconsin, United States; d University of Wisconsin, Department of Medical Physics, Madison, Wisconsin, United States; e Thayer School of Engineering at Dartmouth, Hanover, New Hampshire, United States; f Leibniz Institute of Photonic Technologies, Germany and Member of Leibniz Health Technologies, Jena, Germany; g Friedrich-Schiller-University, Institute of Physical Chemistry and Abbe Center of Photonics, Jena, Germany; h University of California, National Center for Interventional Biophotonic Technologies, Davis, California, United States; i University of California, Department of Neurological Surgery, Davis, California, United States; j University of California, Department of Biomedical Engineering, Davis, California, United States; k University of California, Center for Precision Medicine and Data Science, Davis, California, United States; l University of California, Department of Physiology and Membrane Biology, Davis, California, United States

**Keywords:** digital twins, virtual histology, autofluorescence, uncertainty quantification, metabolic imaging, radiation therapy verification, Cherenkov imaging, Raman spectroscopy, continuous monitoring, personalized surgery

## Abstract

**Significance:**

Digital twins are transitioning from conceptual models to operational frameworks that link measurement, prediction, and intervention in biomedicine. However, most biomedical digital twin efforts remain fragmented, with limited integration across biological scales, sensing modalities, and clinical decision points. Biophotonics provides a uniquely suited measurement foundation for biomedical digital twins by enabling quantitative, physics-grounded, and longitudinal noninvasive measurements spanning molecular, cellular, tissue, organ, and whole-body scales. These capabilities position photonics as a foundational measurement layer for next-generation biomedical digital twins.

**Aim:**

To synthesize current advances and future opportunities in optical digital twins and to establish a unifying framework for how photonic sensing can support digital twin architectures for disease diagnosis, therapy guidance, prevention, continuous monitoring, and interventional healthcare.

**Approach:**

This white paper summarizes perspectives presented at the annual meeting of the international society for optics and photonics (SPIE Photonics West), in the session “Digital Twins as New Approach Methodologies (NAMs) in Biophotonics.” We review five complementary implementations of the digital twin paradigm: (i) virtual tissue staining for histopathology, which combines label-free optical imaging with machine learning-based inference to generate clinically interpretable representations with uncertainty quantification and validation; (ii) cell-level metabolic digital twins that use autofluorescence and redox imaging to predict patient-specific therapeutic responses in tumor organoids and immune cells under controlled perturbations; (iii) therapeutic digital twin frameworks for radiation therapy, in which Cherenkov imaging and radiation chemistry sensing verify treatment delivery and enable biophysical model correction and personalization; (iv) personalized optical digital twins for continuous monitoring that integrate longitudinal photonic sensing with physiological and contextual data to support early detection, prevention, and adaptive care; and (v) personalized digital twins for interventional healthcare.

**Results:**

Across these diverse applications, a common digital twin architecture emerges. Optical measurements define patient state, inference models translate measurements into predictions, therapeutic interventions perturb the system, verification measurements constrain and validate execution, and longitudinal sensing continuously updates the twin over time. The reviewed examples demonstrate that optical measurements can serve as a scalable and biologically relevant data layer linking prediction and intervention across multiple levels of biological organization.

**Conclusions:**

Optical digital twins are no longer merely a conceptual aspiration but are emerging as a practical, measurement-driven infrastructure for precision medicine. The primary challenge is no longer feasibility, but rather the integration, interoperability, validation, and uncertainty quantification of digital twin systems capable of operating safely and at scale. Advances in photonic sensing, computational modeling, and clinical translation position optical digital twins to support real-time, patient-specific clinical decision-making across diagnosis, treatment, monitoring, and prevention.

## 1 Introduction

This perspective paper is intended to summarize the content from the annual meeting of the international society for optics and photonics (SPIE Photonics West), in the session “Digital Twins as New Approach Methodologies (NAMs) in Biophotonics.” The session convened leaders from biomedical engineering, photonics, artificial intelligence, and precision medicine to examine how digital twin technologies are reshaping the future of biophotonics. Through presentations and panel discussions, participants highlighted the transformative potential of digital twins to enable personalized medicine, optimize therapeutic interventions, improve medical device simulation, and advance predictive models of human health across diverse clinical applications. Attention was given to the role of biophotonics in generating high-resolution physiological and molecular data streams that can inform digital twin platforms in real time, creating new opportunities for early disease detection, treatment optimization, and precision intervention strategies. Discussions underscored the importance of interoperability, model validation, transparency, and equitable access to ensure that digital twins are implemented in ways that are trustworthy, reproducible, and clinically actionable. By bringing together expertise spanning biomedical imaging, photonics, artificial intelligence, and computational medicine, the SPIE panel articulated a forward-looking vision in which digital twins become foundational tools for next-generation healthcare.

Digital twins have emerged across science and engineering as an organizational framework to represent, interrogate, and predict the behavior of complex systems. Long utilized in aerospace and advanced manufacturing, the application of digital twins is growing to encompass across disciplines to develop computational constructs that link physical systems to dynamic models through data streams and feedback. As emphasized in the recent National Academies report on digital twins,^1^ the definition of a digital twin is proposed as “A digital twin is a set of virtual information constructs that mimics the structure, context, and behavior of a natural, engineered, or social system (or system-of-systems); is dynamically updated with data from its physical twin; has a predictive capability; and informs decisions that realize value. The bidirectional interaction between the virtual and the physical is central to the digital twin.” A digital twin may range from a tightly coupled, physics-based simulation with real-time data assimilation to a looser federation of models and measurements assembled to support specific decisions. Here, we define an “Optical Digital Twin” as a digital system that is fundamentally characterized by continuous coupling among optical measurements, predictive inference, intervention, and longitudinal state updating through a measurement-constrained feedback loop. The examples reviewed here span a continuum from predominantly data-driven surrogate models to tightly coupled physics-based predictive systems. What unifies these systems is not a single modeling formalism but the persistent coupling among optical measurement, state estimation, prediction, and feedback-driven updating, all constrained by physical observability.

Currently, biomedical implementation of digital twins is fragmented, with limited integration between measurement, modeling, and decision-making.^2^ Importantly, the optical digital twins described here span a continuum from predominantly data-driven surrogate representations to tightly coupled patient-specific physics-based models. In some applications, such as radiation therapy planning and verification, the digital twin includes explicit physical transport simulation and patient-specific parameterization constrained by anatomical and dosimetric measurements. In other settings, including virtual tissue staining and certain image-to-image inference systems, the twin functions more closely as a measurement-constrained surrogate model that maps optical observables onto clinically interpretable image representations through learned transformations. Together, these complementary approaches illustrate how physics-based and AI-driven digital twins can converge to create multiscale modular twins for cancer planning and treatment. Radiation therapy twins can optimize dose delivery and predict treatment response at the organ and tissue level, whereas virtual staining models can provide rapid cellular and molecular characterization of tumor heterogeneity, immune infiltration, and treatment-induced changes. The integration of these modalities could enable continuously updated oncology digital twins that connect imaging, pathology, and therapeutic simulation into a unified framework for precision cancer care, supporting individualized treatment planning, adaptive therapy optimization, and real-time assessment of therapeutic efficacy. Across all implementations, however, the defining feature remains continuous coupling between optical measurement, prediction, and state updating through a physically constrained feedback process.

Biophotonics presents a particularly compelling field that is largely untapped. Optical technologies already span the full translational spectrum, from microscopic and mesoscopic diagnostics to functional and metabolic assessment and real-time monitoring of therapeutic delivery. Historically, biophotonic technologies have evolved as parallel experimental and clinical workflows rather than as components of a unified modeling framework. Within the biophotonics ecosystem, there is an enormous opportunity for the nascent arrival of digital twins to integrate multimodal data streams across biological scales, from proteins to cells, tissues, organs, organ systems, and ultimately clinical phenotypes for real-time clinical decision support.

Photons offer not only rich, multiscale measurements of tissue structure and function but also a well-characterized physical basis to link photonic measurements to forward models of light–tissue interaction. Examples of forward models underlying optical digital twins include radiative transfer equation (RTE)-based light transport, diffusion approximations, Monte Carlo photon transport simulations, fluorescence lifetime decay models, and spectroscopic absorption and scattering models that map tissue composition and physiology onto observable optical signals. These forward models provide physically interpretable links between latent biological state variables and measured optical observables, enabling direct comparison between predicted and measured signals during state estimation and longitudinal updating.

Optical measurements directly define patient state through quantification of physiologically meaningful variables, including tissue structure, oxygenation, perfusion, and metabolic activity, rather than relying on population-derived surrogates. As optical measurements are acquired longitudinally or in real time, optical data define patient state through direct measurement of tissue structure, function, and metabolism, and subsequently constrain model evolution by forcing agreement between predicted and observed light–tissue interactions over time. As additional optical data are collected in time or under varying conditions (e.g., during disease progression, treatment planning, interventional procedures, or therapy delivery) the model states and parameters can be updated to extend the context of use. Model evolution can be constrained by repeated comparison between predicted and measured light–tissue interactions, using known optical physics. Photonics provides measurements with known physical relationships to tissue properties, enabling direct comparison between predicted and measured optical signals and correction of the model when those predictions fail.

The modalities emphasized in this perspective were selected because they collectively demonstrate complementary functional roles required for optical digital twins across biological scales and clinical contexts. Raman spectroscopy provides molecular and biochemical specificity through label-free characterization of chemical composition and cellular state. Autofluorescence and fluorescence lifetime imaging (FLIM) enable estimation of metabolic dynamics, redox balance, and cellular functional state through endogenous fluorophore signatures. Optical coherence tomography (OCT) contributes mesoscopic structural and morphological information with high spatial resolution, supporting the assessment of tissue architecture and microenvironmental organization. Cherenkov imaging provides real-time verification of therapeutic delivery and dose deposition during radiation therapy, enabling measurement-constrained validation of treatment execution. Together, these modalities illustrate how optical measurements can supply complementary biochemical, functional, structural, and therapeutic observability within a unified digital twin framework.

We emphasize that this paper is not intended to provide an exhaustive survey of all optical modalities relevant to biomedical digital twins. Rather, this perspective paper, informed by a SPIE Photonics West 2026 session (18 January 2026), examines the current state of optical digital twins and discusses the future where biophotonic instruments inform clinical action for disease diagnosis, therapy, and prevention.

Additional technologies including photoacoustic imaging, hyperspectral imaging, diffuse correlation spectroscopy, quantitative phase imaging, and emerging multimodal photonic systems, represent important future extensions of the optical digital twin ecosystem. These approaches may further expand the ability of digital twins to capture vascular, metabolic, biomechanical, and physiological state variables across scales while maintaining physically grounded links between measurement and biological interpretation.

## 2 Virtual Tissue Staining as a Data-Driven Digital Twin for Histopathology

The principles outlined above can be observed in key advances in histopathology, in which label-free virtual tissue staining shows the application of data-driven digital twins even when explicit physical forward models are difficult or impossible to construct. The central challenge addressed by this work is the conversion of label-free optical contrast into images that digitally replicate conventional histochemical stains. The mapping between these representations cannot be captured by a simple, reliable set of equations, because it depends on many interacting biological and chemical factors, such as tissue composition, intrinsic fluorescence, cellular structure, and staining chemistry that vary across samples and are not easy to quantify accurately. Supervised deep learning, on the contrary, enables approximation of an unknown function directly from paired image data, positioning virtual staining as an early and operational form of a digital twin for tissue interpretation.^3^

In the conventional pathology workflow, thin tissue sections require chemical staining to generate diagnostic contrast. Even standard stains such as hematoxylin and eosin (H&E) require a couple of hours of processing, whereas immunohistochemical (IHC) stains may require a full day and incur substantial cost. Label-free tissue sections, while optically rich, appear transparent under brightfield microscopy and therefore remain diagnostically inaccessible without staining. Virtual staining replaces the chemical process with a cross-modality transformation.^4^ Autofluorescence microscopy, acquired at a diffraction-limited resolution using endogenous contrast alone, provides the physical measurements. Deep neural networks transform these measurements into virtual equivalents of brightfield or fluorescence-stained slides that match conventional histology in appearance and diagnostic content. Once trained, the model can be applied to previously unseen patient specimens and produces virtual stains in under 1 min on standard computational hardware.

This approach extends beyond simple stains. Virtual staining supports multiplexing on a single tissue section, enabling multiple stains to coexist on the same spatial grid without loss of tissue or registration errors.^4^^,^^5^ For example, it can generate virtual equivalents of Jones silver stain, Masson trichrome, and immunohistochemical markers such as HER2 IHC, using only label-free autofluorescence data acquired through standard filter sets available on digital pathology scanners. Virtual staining results demonstrate that endogenous optical signals encode nanoscale biochemical and structural information that deep learning models can extract and re-express in diagnostically familiar forms.^5^^,^^6^ Importantly, this transformation does not require tissue destruction, repeated sectioning, or antibody incubation. As a result, it saves time and costs and preserves tissue for additional molecular analysis.

Quantitative evaluations against histochemically stained ground truth images showed no statistically significant difference in stain quality or diagnostic accuracy when expert pathologists reviewed virtually stained versus conventional histology slides. In controlled studies, diagnosticians achieved equivalent diagnostic outcomes without knowing whether images originated from chemical or virtual staining pipelines. Virtual staining also has some systematic advantages.^7^ Neural network outputs exhibit consistent staining characteristics across samples and institutions, reducing variability introduced by manual processing and reagent differences, a benefit to both human interpretation and downstream computational analysis using AI.

At the same time, virtual staining exposes a critical failure mode for data-driven digital twins in pathology. Models that produce visually convincing images can also produce visually convincing errors. Experiments demonstrate that expert pathologists may fail to detect induced false structures when no physical ground truth accompanies the synthesized image. Although unrealistic artifacts, such as tissue tears or dust particles, are detectable and clinically manageable, the danger lies in realistic hallucinations, in which a virtually stained image remains internally consistent and interpretable but does not correspond to the measured tissue. In this scenario, a model effectively replaces patient-specific structure with a plausible but wrong alternative, which expert review fails to identify.

Importantly, diagnostic equivalence for optical digital twins cannot be defined solely through visual similarity or pathologist agreement. Although concordance with expert interpretation remains essential, clinically meaningful validation additionally requires quantitative assessment using metrics such as sensitivity, specificity, receiver operating characteristic (ROC) analysis, area under the curve (AUC), reproducibility across imaging sites and instruments, and concordance with established clinical or pathological gold standards. For predictive digital twins, evaluation may further extend to outcome-based metrics, including treatment response prediction, recurrence risk stratification, progression-free survival, and longitudinal monitoring accuracy. These considerations are particularly important for data-driven inference systems such as virtual staining, in which visually plausible outputs may not necessarily correspond to biologically or clinically accurate state estimation. Future optical digital twin validation frameworks will therefore likely require integrated assessment of measurement fidelity, uncertainty quantification (ideally model-free), predictive performance, and clinical utility across diverse patient populations and deployment settings.^8^^,^^9^

The risk of realistic hallucinations forecasts a requirement for digital twins in histopathology.^8^ Virtual staining output images must remain anchored to an underlying physical specimen through measurement-consistency checks between the raw optical data and the synthesized output, enabling autonomous detection of realistic hallucinations across models and institutions without access to ground truth images or the specific virtual staining model.

Virtual tissue staining serves as an important test case for digital twin governance in medicine. It demonstrates that data-driven inference can replace slow/tedious, costly, and destructive laboratory processes while matching clinical needs. It also shows that trust requires persistent linkage between inference and raw measurements, with explicit uncertainty reporting, and model-agnostic validation. In this sense, virtual staining functions as both a successful application and a cautionary roadmap for digital twins in biophotonics.

Uncertainty propagation across biological scales remains a central open challenge for optical digital twins. In practice, uncertainty may originate from measurement noise, adversarial attacks, segmentation variability, biological heterogeneity, incomplete observability, and mismatch between idealized forward models and biological reality.^8^ These uncertainties propagate through hierarchical inference pipelines as estimates derived from cellular measurements influence tissue-level interpretations, which subsequently constrain organ-level and longitudinal patient-state predictions. Future optical digital twins will therefore likely require probabilistic state-estimation and defense frameworks, Bayesian inference, ensemble modeling, and uncertainty-aware data assimilation approaches that continuously updating confidence estimates as new measurements become available across scales and time.

## 3 Cell-Level Metabolic Digital Twins for Therapy Response Prediction

The same principles that govern tissue-scale digital twins apply at the cellular scale, in which optical measurements enable cell-level metabolic screening to predict functional response to therapy. The clinical problem addressed is the challenge to predict treatment efficacy for individual cancer patients. Hundreds of therapeutic options may apply to a given diagnosis, and current clinical practice relies on sequential trial-and-error approaches that expose patients to toxicity and delay effective treatment. A cell-level digital twin aims at direct measurement and prediction of an individual cellular response to candidate therapies prior to treatment.

The core strategy is patient-derived tumor organoids as experimental proxies for *in vivo* tumor behavior.^10^ Organoids preserve three-dimensional architecture and cellular heterogeneity and allow for controlled perturbation. Drug exposure serves as a defined input, and optical measurements quantify the resulting cellular response. The selected readout targets metabolism, which constrains cell proliferation and survival across cancer types. Metabolic activity reflects the availability of energy and biosynthetic precursors required for division, making it a sensitive indicator of therapeutic effect.

Two-photon autofluorescence microscopy allows direct measurement of endogenous fluorophores, primarily NADH, NADPH, and FAD, in metabolic reactions, and these fluorophores emit fluorescence that encodes both concentration and binding state.^11^^,^^12^ Fluorescence lifetime measurements resolve free and protein-bound states of endogenous fluorophores, enabling extraction of multivariate metabolic features from individual cells. Combined with intensity-based optical redox ratio measurements, this approach yields a high-dimensional description of cellular metabolic state. Early studies demonstrate that these features predict treatment response across preclinical models and clinical patient samples.

Although two-photon lifetime imaging establishes biological validity, it imposes practical limitations. The approach remains low throughput, instrument-intensive, and difficult to deploy widely in clinical or translational settings. To address this constraint, the work transitions to widefield optical redox ratio imaging on standard epifluorescence microscopes. This approach trades per-cell lifetime resolution for speed, scalability, and accessibility. Patient-derived organoids grow in multiwell plates, and intensity-based redox measurements support rapid screening across many drug conditions.

Robust deployment at this scale requires careful algorithmic control. Individual organoids exhibit distinct growth patterns and metabolic trajectories, so analysis tracks each organoid over time rather than relying on population averages. Segmentation algorithms isolate organoids from background, compensate for illumination variation, and account for sample heterogeneity. Analysis focuses on the proliferative rim of each organoid, in which the metabolic response most directly reflects the treatment effect, rather than the hypoxic or necrotic core. Longitudinal tracking over days captures dynamic response rather than static snapshots. These methods enable the prediction of patient-specific responses to standard therapies, including chemotherapy, targeted antibodies, and radiation.

The framework also extends naturally to immunotherapy, in which predictive tools remain especially limited. Chimeric antigen receptor (CAR) T cell therapy relies on *ex vivo* manipulation and expansion of patient T cells before reinfusion.^13^ Treatment success depends strongly on T cell metabolic state, which governs cytotoxic potency versus exhaustion. Optical metabolic imaging distinguishes these states by measuring endogenous fluorescence signatures associated with energy production and biosynthetic capacity.

Experimental manipulation of T cell metabolism produces populations with high or low therapeutic potency. Label-free optical measurements predict which T cell products will eradicate tumors *in vivo* prior to administration. This introduces a pretreatment decision point that avoids ineffective or harmful interventions. To support clinical scalability, the work develops an autofluorescence lifetime flow cytometer capable of resolving NADH lifetime signatures at high throughput.^14^ Engineering advances enable sufficient photon collection from weak endogenous signals to fit multiexponential decay models, and benchmarking confirms agreement with two-photon microscopy.

The progression from detailed cellular measurement to scalable screening platforms defines a complete cell-level digital twin. The twin is initialized from patient-derived cells, accepts controlled perturbations, measures metabolic responses, and predicts therapeutic outcomes within clinically relevant timeframes. At each stage, the approach exposes limitations tied to biological drift, culture variability, and measurement noise. Trust therefore depends on explicit uncertainty characterization, longitudinal consistency checks, and validated operating regimes.

In the context of optical digital twins, this work demonstrates that metabolism-based photonic measurements can support rapid, patient-specific prediction across both conventional therapies and emerging immunotherapies. It also highlights a central constraint shared with tissue-level twins: increased measurement throughput does not substitute for explicit validity limits. A cell-level twin remains clinically actionable only when it reports when predictions fall outside calibrated biological or instrumental bounds.

## 4 Radiation Therapy Digital Twins for Treatment Planning, Verification, and Biological Correction

Extending the digital twin framework from cellular response prediction to physical intervention delivery leads naturally to radiation therapy, in which patient-specific models have always guided personalized treatments to individual patients. However, the experience in this application of digital twins helps illustrate a key point, in that there needs to be end-to-end validation of the digital twin for it to be effective, meaning verification of delivery is needed at the point of care. Radiation therapy represents one of the most mature and widely deployed digital twin systems in clinical medicine. Treatment planning relies on a detailed patient-specific computational model derived from CT, MRI, and PET imaging to simulate radiation transport and predict dose deposition with millimeter-scale precision. Inverse planning algorithms optimize beam geometry and intensity to maximize dose conformality to tumor volumes while minimizing exposure to surrounding tissue. This planning process constitutes a digital representation of the patient that guides therapy for tens of thousands of patients each day.

Despite the technological sophistication of extensive planning of the treatment, a fundamental limitation remains. Radiation dose cannot be directly measured inside patient tissue during treatment. Clinical practice relies on extensive simulation and linac QA, rather than verification of delivery. As treatment complexity increases through intensity-modulated photon therapy, image-guided therapy, and the growing adoption of proton therapy, any gap between planned dose and delivered dose becomes increasingly consequential. Daily patient setup must achieve submillimeter accuracy over multiweek treatment courses, often in patients with limited mobility or changing anatomy. Even small deviations in positioning, motion, or beam alignment can invalidate assumptions embedded in the treatment plan.

This limitation defines the role of optical technologies within the radiation therapy digital twin. Cherenkov imaging provides real-time visualization of radiation beam delivery by detecting faint optical emission generated as charged particles traverse tissue^15^^,^^16^ High-sensitivity intensified CMOS camera systems, combined with fast signal processing, enable single-photon-level imaging under full ambient lighting conditions.^17^^,^^18^ These systems operate in clinical environments and capture beam position and shape during treatment delivery. Cherenkov images expose misalignment, motion-induced error, and day-to-day variability that remain invisible to the treatment planning system alone.

The approach scales beyond localized treatments. Whole-body Cherenkov imaging enables surface dose mapping for patients requiring total skin electron therapy, such as those with mycosis fungoides.^19^ Optical measurements register dose delivery across large anatomical areas and support construction of spatially resolved dose maps. These maps function as a measurement-based overlay on the digital twin, confirming coverage, and identifying deviations from intended delivery. Optical verification methods additionally support quantitative assessment of treatment delivery accuracy relative to planned dose distributions. Cherenkov imaging systems can detect beam alignment deviations at millimeter-scale precision, assess spatial consistency of delivered surface dose, and provide comparison against conventional dosimetry measurements and treatment planning system predictions. These measurements enable identification of motion-induced error, setup variability, and deviations between planned and delivered treatment geometry. In this framework, optical verification serves as a measurement-constrained validation layer that continuously tests the agreement between predicted and executed therapy, rather than relying exclusively on pretreatment simulation assumptions.

Inverse problems in optical digital twins are frequently ill-posed because measured optical signals often arise from multiple interacting biological and physical parameters. Stable recovery of latent biological states therefore requires regularization through the incorporation of prior information and measurement constraints. Depending on the application, these priors may include known optical physics, spatial smoothness assumptions, tissue anatomical structure, learned latent representations, multimodal consistency constraints, or longitudinal temporal continuity. In many cases, repeated measurements acquired under perturbation or over time further constrain the solution space and improve the robustness of inferred state estimates.

Current research efforts extend the digital twin beyond geometric verification toward biological relevance. Dose alone does not determine treatment outcome. Biological response depends on tissue heterogeneity, oxygenation, radiation type, and dose rate. Proton therapy introduces variable relative biological effectiveness across tissue and energy spectra. Hypoxia alters radiation sensitivity, and ultra-high dose rate (FLASH) therapy introduces additional chemical and temporal effects.^20^^,^^21^ Incorporating these factors into a digital twin requires new forms of measurement rather than additional simulation complexity alone. Radiation chemistry provides a pathway for such measurement, and optical probes are some of the most reliable tools for this. Ionizing radiation initiates water radiolysis and generates short-lived reactive species. Almost most intermediates decay rapidly, a limited set persists long enough for optical detection. Recent work demonstrates that free electrons produce a measurable absorption feature near 700 nm, enabling *in vivo* estimation of radiation chemical dose in FLASH.^21^ Fiber-optic probes detect this transient absorption during irradiation, providing direct evidence of deposited chemical energy inside tissue. This development introduces a new validation layer that can link physical dose delivery to biological effect through direct validation of the dose delivered inside tissue.

Within the broader context of optical digital twins, radiation therapy illustrates a critical principle. A digital twin achieves clinical value only when it remains coupled to measurement during execution, not only during planning. This end-to-end use is critical, where the twin is created and validated through its use. Photonics supplies the missing observability required to verify delivery, quantify deviation, and support future extensions toward biology-aware radiation therapy.

## 5 Personalized Optical Digital Twins for Continuous Health Monitoring and Intervention

Although radiation therapy digital twins emphasize measurement during therapeutic execution, personalized optical digital twins extend the same measurement-driven coupling across time, shifting the digital twin paradigm from episodic intervention toward continuous health monitoring, prevention, and adaptive care. This perspective reframes the digital twin not as a tool for isolated clinical decisions, but as a longitudinal, patient-specific reference system that evolves with the individual. This perspective is informed by a European public–private initiative coordinated through Photonics21^22^ and reflects a broader need to shift healthcare from reactive treatment toward proactive, measurement-driven health management. Current systems emphasize reactive treatment after disease onset, whereas demographic aging, chronic disease prevalence, pandemics, and rising healthcare costs expose the limitations of this model. A personalized optical digital twin aims to support proactive health management through individualized, real-time, and noninvasive measurement.

The core premise is that health status can be represented as a dynamic, patient-specific reference state derived from repeated optical measurements. Deviations from this reference state indicate early disease processes or altered physiological function before clinical symptoms appear. To achieve this goal, measurement technologies must be developed that operate continuously or frequently, impose minimal burden, and capture molecular and biochemical information rather than coarse physiological signals alone. In contrast to conventional diagnostic thresholds derived from population averages, this reference state is individualized and history-aware. Health is therefore defined relative to a person’s own longitudinal trajectory rather than deviation from an external norm. This distinction is essential for early detection, as many pathological processes manifest first as subtle personal deviations long before crossing population-based clinical thresholds.

Optical technologies uniquely satisfy the requirements of longitudinal digital twins. They enable nonionizing, repeatable measurements; provide direct access to molecular and biochemical information; and scale from laboratory instruments to point-of-care, home, and wearable systems. Crucially, optical measurements remain physically interpretable through established light–tissue interaction models, allowing digital twins to remain constrained by measurement rather than model confidence alone. Advances in photonic integration and device scaling allow migration from laboratory instruments toward compact, robust systems suitable for decentralized care. These properties position photonics as a foundation for everyday health monitoring rather than episodic clinical testing.

One representative example focuses on blood-based diagnostics.^23^^–^^25^ Conventional blood counts report cell numbers and basic parameters but provide limited biochemical insight. Optical spectroscopy enables access to molecular fingerprints associated with pathogens, cellular damage, metabolic state, and immune activation. Raman, infrared, and related spectroscopic techniques extract chemical information from blood cells and extracellular vesicles without labels. This information supports deeper characterization of health status than cell counts alone. Beyond blood-based diagnostics, Raman spectroscopy has also demonstrated significant potential for therapy-response monitoring, immune-cell phenotyping, infection diagnostics, and intraoperative tissue characterization. Owing to its ability to provide label-free molecular information across cellular, tissue, and organ levels, Raman spectroscopy represents a particularly attractive sensing modality for future multiscale optical digital twins, linking molecular phenotypes with patient-specific disease trajectories and therapeutic outcomes.

Recent work demonstrates this approach using microliter-scale blood samples obtained by finger prick.^25^ Microfluidic chip devices separate red blood cells, white blood cells, and platelets. Integrated Raman spectroscopy acquires chemical signatures from individual cell populations. Machine learning methods classify cell types and infer activation states from these spectra. Miniaturized Raman systems enable a sample-to-answer workflow that supports individualized biochemical state estimation within clinically relevant timeframes. Within a digital twin framework, these measurements initialize and update patient-specific state variables rather than serving as isolated diagnostic readouts. Their value lies not in single measurements, but in their repeated use to track deviation, recovery, and response over time.

Cross-modal consistency within optical digital twins is maintained through shared latent physiological and biochemical state variables that simultaneously constrain multiple optical observables. For example, tissue oxygenation, metabolism, perfusion, cellular composition, and structural organization may jointly influence Raman spectra, fluorescence lifetimes, OCT scattering signatures, and diffuse spectroscopic measurements. Rather than interpreting each modality independently, multimodal digital twins integrate complementary observables into unified physiological state estimates constrained by both optical physics and biological consistency relationships.

A personalized optical digital twin integrates such photonic measurements with complementary data sources. Wearable sensors contribute physiological parameters such as heart rate and activity. Environmental exposure, lifestyle factors, and social context provide additional modifiers of the health state. Computational models combine these inputs to simulate organ function, immune response, and disease trajectories. The resulting twin defines a patient-specific baseline rather than a population norm, allowing detection of deviation relative to an individual’s own historical data. This framework supports several functions. Continuous monitoring enables early detection of disease before symptom onset. Longitudinal tracking informs therapy response when treatment occurs. Decision-support systems generate individualized recommendations based on measured deviation and predicted progression. Both clinicians and patients receive interpretable assessments of the current state and projected risk. Within this architecture, photonic measurements provide the molecular and biochemical ground truth that anchors higher-level physiological and contextual data streams.

The approach also extends to intraoperative and interventional settings, in which diagnosis and therapy merge. Multimodal optical imaging combines nonlinear Raman techniques, two-photon excited fluorescence, fluorescence lifetime imaging, and second harmonic generation to capture chemical, structural, and functional information simultaneously. Deep learning methods translate these multimodal measurements into representations familiar to clinicians, including virtual histology comparable to hematoxylin and eosin staining. Endomicroscopic systems support three-dimensional imaging of tissue in near real time.

Several challenges remain central to deployment. First, longitudinal digital twins amplify the impact of systematic bias and model drift, as errors can accumulate over time. Second, artificial intelligence systems must remain transparent and uncertainty-aware; confident but incorrect predictions undermine trust more severely than acknowledged uncertainty. Third, usability, data security, and robustness must be addressed simultaneously, as failure in any one-dimension compromises clinical adoption. These constraints reinforce the need for governance structures that treat digital twins as continuously validated systems rather than static medical devices. Systems must meet requirements for usability, security, and scalability across clinical and home environments. Progress will depend on tightly coordinated contributions from research institutions, industry, clinical partners, policymakers, and the public. Investment spans fundamental research, pilot manufacturing, clinical translation, ecosystem development, and workforce training. The goal is a durable healthcare infrastructure that supports early detection, decentralized care, and long-term system resilience. In this sense, personalized optical digital twins provide the temporal backbone that connects diagnostic, therapeutic, and verification-focused twins into a coherent, lifelong health model.

## 6 Personalized Digital Twins for Interventional Healthcare

**Fig. 1:**
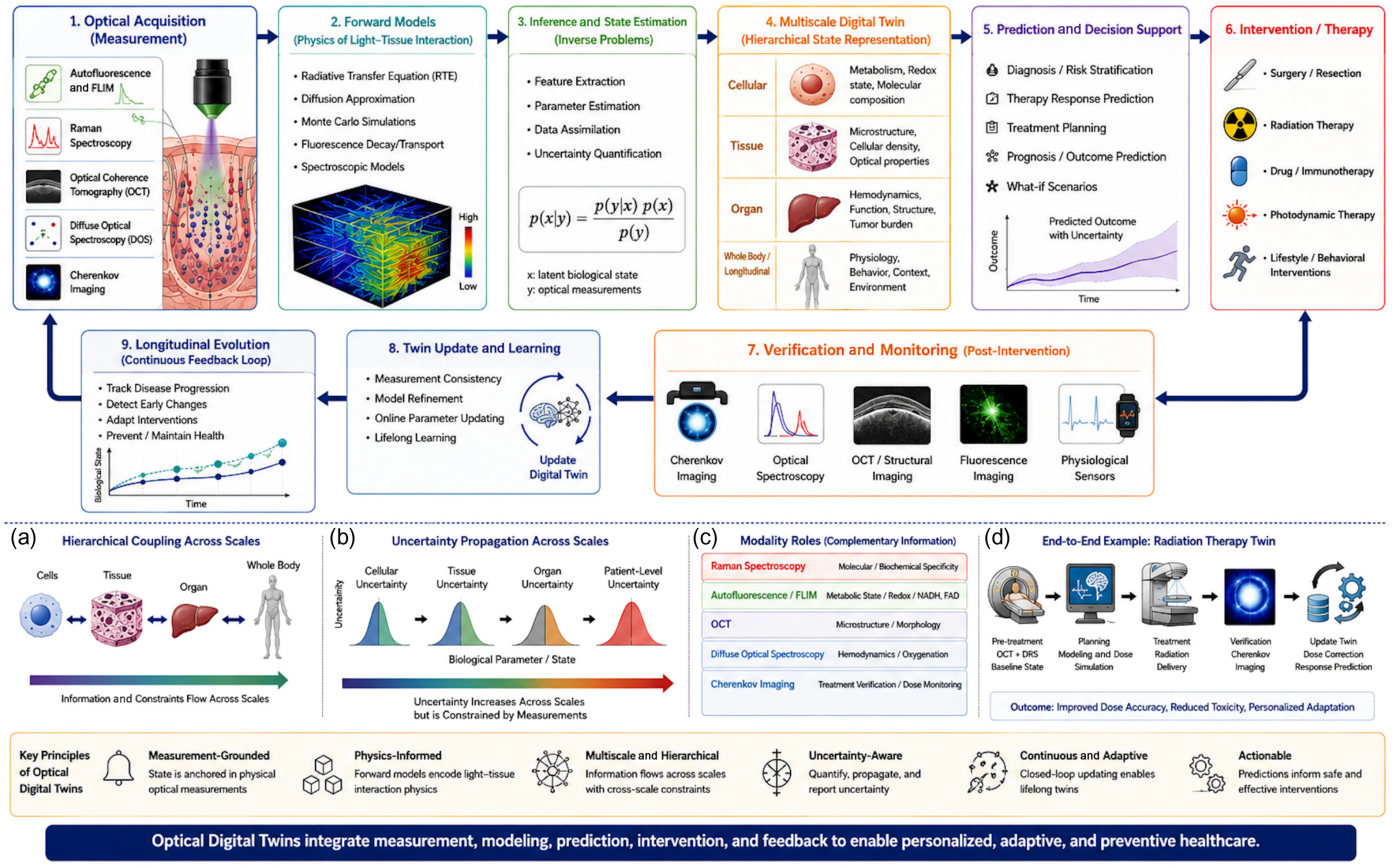
Unified architecture of optical digital twins across biological scales. This schematic illustrates a measurement–inference–prediction–intervention–verification feedback loop for constructing and continuously updating optical digital twins. (1) Multimodal optical measurements, including autofluorescence and fluorescence lifetime imaging microscopy (FLIM), Raman spectroscopy, optical coherence tomography (OCT), diffuse optical spectroscopy (DOS), and Cherenkov imaging, provide quantitative observations of tissue structure, composition, and function. (2) Physics-based forward models describing light–tissue interactions, including radiative transfer, diffusion approximations, Monte Carlo simulations, fluorescence transport, and spectroscopic models, map latent biological states to observable optical signals. (3) Inverse modeling and state estimation integrate measurements with prior knowledge to infer biological parameters, perform data assimilation, and quantify uncertainty. (4) Inferred states are represented across hierarchical biological scales—from cellular metabolism and molecular composition to tissue microstructure, organ function, and whole-body physiology—forming a multiscale digital twin. (5) The digital twin generates predictions and supports clinical decision-making, including diagnosis, risk stratification, treatment planning, therapy response assessment, and prognosis while explicitly propagating uncertainty. (6) Predicted outcomes inform therapeutic interventions such as surgery, radiation therapy, drug or immunotherapy, photodynamic therapy, and behavioral modifications. (7) Postintervention monitoring using optical and physiological measurements verifies outcomes and provides new observations for model refinement. (8) Continuous learning updates model parameters, improves consistency between predictions and observations, and enables lifelong adaptation of the digital twin. (9) Longitudinal tracking captures disease progression, treatment effects, and evolving patient-specific states, completing the feedback loop. The lower panels highlight key design principles: (a) hierarchical coupling across cellular, tissue, organ, and whole-body scales; (b) propagation of uncertainty across biological levels; (c) complementary information provided by different optical modalities; and (d) an example radiation therapy digital twin workflow integrating pretreatment imaging, treatment planning, radiation delivery, optical verification, and model updating. Together, these components establish a physics-informed, uncertainty-aware framework for personalized, adaptive, and preventive healthcare enabled by optical digital twins.

A shared theme across recent work on digital twins^26^ and surgical data science^27^ is the transition from static, retrospective planning tools to operational, measurement-driven systems that continuously couple real-time data streams to predictive models during intervention. In the surgical setting, for example, a personalized digital twin is increasingly framed as a dynamic virtual construct that represents individual patient anatomy and physiology across the perioperative continuum, updating as new measurements are acquired and enabling adaptive decision support in the operating room. As illustrated in Fig. 1, interventional digital twins represent a natural extension of the measurement–inference–prediction–intervention–verification framework that underpins optical digital twins across biological scales. In this setting, optical measurements acquired during intervention continuously update patient-specific state estimates, enabling adaptive decision-making and real-time validation of therapeutic actions.^28^^,^^29^ This formulation aligns closely with the architecture emerging in optical digital twins more broadly: measurements define patient state, inference translates signals into clinically interpretable representations, interventions perturb the system, and verification measurements constrain execution and update the twin over time.

As digital twins evolve from passive data aggregators into more agentic and adaptive systems, their clinical value depends on maintaining bidirectional coupling between the physical and virtual domains. Surgical twins therefore require not only preoperative imaging and simulation, but intraoperative observations that can detect deviation from predicted tissue state and guide real-time correction. This requirement mirrors challenges identified in virtual tissue staining, in which visually plausible inference must remain anchored to the underlying tissue specimen to avoid hallucinated structure, and in metabolic digital twins, in which endogenous fluorescence signatures provide functional readouts of therapy response under controlled perturbations. In surgery, the analogous need is continuous validation of tissue type, viability, and malignancy at the resection margins during intervention, rather than reliance on static anatomical assumptions.

Within this landscape, photonic-based technologies emerges as a uniquely enabling measurement substrate for surgical digital twins because they can provide quantitative, label-free, and physically grounded pathophysiological or functional features at the point of care. Optical imaging and sensing modalities such as fluorescence-guided imaging including lifetime imaging, Raman spectroscopy, optical coherence tomography, and diffuse optical spectroscopy can extend image-guided surgery beyond anatomical navigation toward molecular and metabolic state estimation in real-time. These techniques can support intraoperative virtual histology-like representations of tissue, functional assessment of perfusion and metabolism, and detection of residual cancer and biochemical heterogeneity at surgical margins, all of which directly impact resection strategy. In this way, optical guidance becomes not merely an adjunct visualization tool, but a verification and update layer that closes the loop between model prediction and surgical execution, analogous to Cherenkov-based verification in radiation therapy digital twins. This integrated framework is currently being pursued under the umbrella of the NIH–NIBIB National Center for Interventional Biophotonics Technologies (NCIBT) at UC Davis.^22^

More broadly, the surgical data science paradigm calls for systematic capture and integration of heterogeneous intraoperative and longitudinal data streams to support quantifiable, data-informed intervention. Biophotonic technologies are particularly aligned with this vision because they generate high-dimensional, information-rich measurements that can be fused with preoperative imaging, physiological monitoring, and contextual clinical data to construct continuously updated, uncertainty-aware surgical twins. Taken together, these perspectives point toward a future in which optical digital twins provide the measurement-driven infrastructure for personalized, adaptive surgery: photons define tissue state at multiple biological scales, inference models translate optical signals into actionable representations, interventions are guided by real-time feedback, and longitudinal sensing ensures that the twin remains constrained by the evolving biology of the individual patient.

### 6.1 Physics-Constrained State Estimation and Multiscale Integration

Optical digital twins can be formally viewed as hierarchical state-estimation systems in which optical measurements constrain latent biological variables across multiple spatial and temporal scales. In this framework, forward models describe the relationship between tissue state and observable optical signals through light–tissue interaction physics, including radiative transfer, diffusion approximations, Monte Carlo photon transport, fluorescence lifetime decay, scattering, absorption, and spectroscopic models. These models provide physically interpretable mappings between biological structure or function and measured optical observables.

Inverse inference maps measured optical signals onto latent biological parameters such as metabolism, oxygenation, perfusion, tissue viability, cellular composition, or therapeutic response. In many applications, these inverse problems are partially identifiable and intrinsically ill-posed because multiple biological states can generate similar optical measurements under realistic acquisition conditions. Practical implementations, therefore, require regularization through biophysical priors, multimodal constraints, longitudinal consistency, perturbation experiments, and uncertainty-aware inference frameworks. Importantly, the examples reviewed here span a continuum from predominantly data-driven surrogate models to tightly coupled physics-based twins; however, all share the defining characteristic of continuous measurement-constrained updating through a closed feedback loop linking prediction, intervention, verification, and model refinement.

Within this hierarchy, cellular-, tissue-, organ-, and patient-level twins are not independent systems, but coupled representations that exchange information and constraints across scales. Cellular metabolic measurements may inform tissue-level viability estimates, whereas organ-level physiological models constrain interpretation of local optical signatures. Similarly, multimodal optical measurements provide complementary observability of shared latent biological states. Raman spectroscopy contributes molecular specificity, autofluorescence lifetime imaging reports metabolic dynamics, optical coherence tomography captures mesoscopic structural organization, and Cherenkov imaging provides treatment verification during therapeutic delivery. Consistency across modalities is therefore maintained through shared physiological state representations rather than independent interpretation of each modality in isolation.

A central challenge for future optical digital twins lies in uncertainty propagation and model mismatch across scales. Measurement noise, segmentation variability, incomplete observability, biological heterogeneity, and departures from idealized forward models can all introduce uncertainty into inferred biological states. As digital twins evolve longitudinally, these uncertainties may accumulate unless constrained by repeated measurement updating and cross-modal validation. Accordingly, clinically deployable optical digital twins will likely require probabilistic state estimation, uncertainty-aware inference, and continuous recalibration strategies capable of identifying when predictions extend beyond validated operating regimes. These challenges remain open research frontiers and define critical directions for the future development of trustworthy and interoperable biomedical digital twin systems.

## 7 Conclusions

Within the biophotonics ecosystem, there is a substantial opportunity for digital twins to integrate multimodal data streams across biological scales, from proteins to cells, tissues, organs, organ systems, and ultimately clinical phenotypes for real-time clinical decision support. The four topics described in this perspective paper demonstrate that this integration is already underway as a set of complementary implementations that collectively define the architecture of an optical digital twin. The future has the potential to allow for integration into a single unified system.

At the molecular and subcellular scale, endogenous optical signals parameterize biochemical state through fluorescence intensity and lifetimes, Raman spectra, absorption coefficients, and nonlinear contrast responses. These observables depend on metabolic flux, protein binding, and chemical composition and require no exogenous labels. Cell-level digital twins use these measurements to estimate functional response under controlled perturbations, including drug exposure and immune activation. This formulation enables rapid prediction of therapeutic response and explicitly defines the subset of measurement and state space for which model predictions remain statistically identifiable and physically consistent given biological variability and measurement noise.

At the tissue scale, label-free optical imaging measures spatial structure, heterogeneity, and microenvironmental context. Virtual tissue staining maps these measurements to clinically interpretable representations through learned transformations constrained by optical measurements and physics. This mapping defines the interface between measurement space and diagnostic space and requires continuous consistency with the underlying optical signal to avoid unobserved inference errors or hallucinations. Tissue-scale digital twins estimate structural properties of tissue, including spatial organization, cellular boundaries, and microenvironmental heterogeneity from indirect, label-free optical measurements rather than from direct physical labeling. In many clinical and *in vivo* settings, destructive validation methods such as chemical staining or repeated biopsies are limited in availability, and therefore, a direct physical ground truth might not exist against which inferred structures can be checked. Under these conditions, the digital twin must explicitly represent uncertainty, identifying regions where structural estimates are weakly constrained by measurements or sensitive to model assumptions, rather than presenting all inferred structure as equally reliable.

Several important challenges remain for the development of clinically deployable optical digital twins. These include the identifiability of latent biological state variables, parameter degeneracy arising from incomplete observability, a mismatch between idealized optical models and heterogeneous biological reality, robustness to measurement uncertainty and segmentation variability, and the determination of the minimal information required for reliable prediction. Addressing these challenges will require multimodal sensing, uncertainty-aware inference, longitudinal validation, adaptive modeling strategies, and continuous integration of new measurements into dynamically evolving patient-specific models.

Importantly, these same principles extend naturally into the domain of personalized interventional healthcare, including image-guided and precision surgery. Interventional digital twins must evolve beyond static preoperative models toward intraoperative, measurement-coupled systems that can validate tissue state and guide adaptive decision-making *in situ* at the point of care. Optical imaging and sensing modalities, including fluorescence-guided imaging, Raman spectroscopy, optical coherence tomography, and diffuse optical spectroscopy, can provide quantitative, label-free, and physically grounded information on tissue malignancy, viability, perfusion, and biochemical heterogeneity directly within the operative field. In this framework, photonics serves not only as a diagnostic modality but also as a critical verification and update layer, enabling closed-loop surgical digital twins that remain anchored to measured tissue state throughout the intervention.

At the organism and population interface, personalized optical digital twins extend state estimation across time. Longitudinal photonic sensing integrates molecular fingerprints, physiological signals, and contextual data to establish patient-specific baselines. Deviations from these baselines inform early detection, therapy response, and recovery monitoring. This temporal integration connects episodic diagnostic and therapeutic twins into a continuous model of health rather than disease alone.

Importantly, the minimal sufficient feature set for an optical digital twin is unlikely to be universal across applications. The required measurements and latent variables depend on the biological scale, disease context, intervention type, and intended clinical decision. In some settings, low-dimensional metabolic or structural measurements may provide sufficient predictive power, whereas other applications may require multimodal integration of biochemical, physiological, anatomical, and longitudinal data streams. Determining which measurements are both necessary and sufficient for robust prediction while minimizing model complexity, acquisition burden, and computational cost remains an active area of investigation for future digital twin development.

Moreover, robustness to measurement noise, segmentation variability, and acquisition artifacts represents a critical requirement for clinically deployable optical digital twins. Optical measurements may be affected by photon noise, motion artifacts, illumination variation, tissue heterogeneity, and imperfect segmentation or registration pipelines. Accordingly, future digital twin systems will likely require explicit robustness testing under realistic or adversarial perturbations, uncertainty-aware inference frameworks capable of reporting confidence intervals for inferred biological states, and out-of-distribution detection mechanisms that identify when measurements fall outside validated training or calibration regimes. These safeguards are especially important in longitudinal and interventional settings, where small systematic errors may accumulate over time and propagate across scales.

Taken together, developing technologies in biophotonics may comprise a single closed-loop system if they can be connected in meaningful ways. Optical measurements acquire state at multiple biological scales. Inference models translate these measurements into interpretable representations and predictions. Therapeutic actions perturb the system. Verification measurements test whether perturbations match the intended behavior. Longitudinal sensing updates the twin as the system evolves. Across all scales, uncertainty quantification governs when predictions support action and when additional measurements are required. Clinical experts remain an explicit component of this loop, using model outputs, uncertainty estimates, and domain knowledge to authorize intervention, override automated recommendations, and determine when measurement, inference, or treatment assumptions no longer hold.

Within a multiscale optical digital twin framework, histology-level, organ-level, and whole-body monitoring systems are best viewed as hierarchically coupled rather than fully independent twins. Cellular and tissue-scale optical measurements provide local biochemical, metabolic, and structural information that can inform organ-level physiological state estimation and therapeutic response prediction. Conversely, organ-level physiology, systemic immune state, vascular dynamics, and longitudinal patient context constrain interpretation of local optical signatures and tissue behavior. In this framework, information flows bidirectionally across scales, enabling local measurements to refine higher-level predictions while larger-scale physiological context constrains lower-scale inference. Establishing mathematically rigorous frameworks for multiscale coupling, uncertainty propagation, and longitudinal updating across these interconnected biological levels remains an important future direction for optical digital twin development.

Longitudinal optical digital twins will additionally require scalable infrastructure capable of supporting multimodal data storage, real-time streaming, longitudinal synchronization, federated learning, and GPU-accelerated inference across heterogeneous clinical environments. As optical digital twins evolve toward continuously updated systems integrating imaging, physiological monitoring, molecular measurements, and contextual patient data, computational demands will increasingly extend beyond standalone analysis pipelines toward interoperable clinical data ecosystems. Efficient data compression, distributed processing, privacy-preserving learning strategies, and uncertainty-aware cloud or edge computing architectures will likely become essential components of future clinically deployable digital twin platforms.

Optical measurements are uniquely poised to underpin digital twins as they combine high information density with physical traceability. They provide a common measurement substrate that spans length scales and clinical contexts while remaining grounded in known light–tissue interactions. This shared physical basis enables consistent uncertainty propagation across modalities and allows digital twins to remain constrained by reality rather than model confidence alone.

Validation of optical digital twins requires application-specific reference standards spanning multiple biological and clinical scales. In histopathology applications, conventional chemically stained tissue sections and pathology consensus interpretation remain the primary gold standards for assessing diagnostic concordance. In radiation therapy, validation may include comparison against treatment planning system predictions, ion chamber measurements, film dosimetry, and *in vivo* dosimeters. Organ- and physiological-level twins may additionally be benchmarked against MRI, PET, CT, ultrasound, or established functional imaging modalities. At the cellular scale, flow cytometry, genomic assays, and molecular profiling provide orthogonal validation of inferred metabolic or immune states. Ultimately, clinically meaningful validation must also incorporate longitudinal patient outcomes, including treatment response, recurrence, toxicity, progression-free survival, and overall survival. These complementary reference standards collectively define the framework through which optical digital twins can be evaluated for both measurement fidelity and clinical utility.

The primary challenge ahead lies in integrating technologies into interoperable systems. Success will depend on designing systems that allow measurement, inference, and decision support to co-evolve rather than compete. In a future state, an integrated form will ensure that optical digital twins provide a path to real-time, patient-specific clinical decision support spanning diagnosis, therapy selection, delivery verification, monitoring, and prevention. The work presented here suggests that the biophotonics community already holds the necessary components. The future task now is to assemble them into a coherent, measurement-driven framework that can operate safely and at scale. If successful, optical digital twins may transform biophotonics from a collection of powerful measurement technologies into a predictive, adaptive, and continuously learning framework for precision healthcare, enabling a future in which diagnosis, intervention, monitoring, and prevention are seamlessly connected through measurement-driven intelligence.

## Data Availability

Data sharing is not applicable to this article, as no new data were created or analyzed.

## References

[1] “Foundational research gaps and future directions for digital twins,” The National Academies Collection: Reports funded by National Institutes of Health (2024). 39088664

[2] Clancy C. E. Landman B. A., “Towards credible digital twins for basic and preclinical research,” Nat. Rev. Methods Primers 6, 5 (2026).2662-8449 10.1038/s43586-025-00454-3

[3] Chen S. et al., “Optical generative models,” Nature 644, 903–911 (2025). 10.1038/s41586-025-09446-5 40866675 PMC12390839

[4] Zhang Y. et al., “Pixel super-resolved virtual staining of label-free tissue using diffusion models,” Nat. Commun. 16, 5016 (2025). 10.1038/s41467-025-60387-z 40447613 PMC12125245

[5] Zhang Y. et al., “Virtual staining of label-free tissue in imaging mass spectrometry,” Sci. Adv. 11, eadv0741 (2025). 10.1126/sciadv.adv0741 40749063 PMC12315973

[6] Zhang Y. et al., “Deep learning-enabled virtual multiplexed immunostaining of label-free tissue for vascular invasion assessment,” BME Front. 7, 0226 (2026). 10.34133/bmef.0226 41676162 PMC12886716

[7] Li Y. et al., “Label-free evaluation of lung and heart transplant biopsies using tissue autofluorescence-based virtual staining,” BME Front. 6, 0151 (2025). 10.34133/bmef.0151 40606521 PMC12217214

[8] Huang L. et al., “A robust and scalable framework for hallucination detection in virtual tissue staining and digital pathology,” Nat. Biomed. Eng. 9, 2196–2214 (2025). 10.1038/s41551-025-01421-9 40523934 PMC12705451

[9] Wang Y. et al., “Universal and transferable attacks on pathology foundation models using microscopic perturbations,” Light Sci. Appl. 15, 2026). 10.1038/s41377-026-02347-w 258. 42225639 PMC13226705

[10] Kratz J. D. et al., “Subclonal response heterogeneity to define cancer organoid therapeutic sensitivity,” Sci. Rep. 15, 12072 (2025). 10.1038/s41598-025-96204-2 40200028 PMC11978853

[11] Datta R. Skala M. C. Miskolci V., “Assessing intracellular metabolism of immune cells in situ in live zebrafish larvae by autofluorescence lifetime imaging microscopy of NAD(P)H and FAD,” Methods Mol. Biol. 2983, 169–192 (2026). 10.1007/978-1-0716-4901-5 41478977

[12] Datta R. et al., “Single cell autofluorescence imaging reveals immediate metabolic shifts of neutrophils with activation across biological systems,” Front. Immunol. 16, 1617993 (2025). 10.3389/fimmu.2025.1617993 40852713 PMC12367685

[13] Pham D. L. et al., “Label-free metabolic imaging monitors the fitness of chimeric antigen receptor T cells,” Nat. Biomed. Eng. 10, 785–802 (2025). 10.1038/s41551-025-01504-7 40958004 PMC12445593

[14] Samimi K. et al., “Autofluorescence lifetime flow cytometry with time-correlated single photon counting,” Cytometry A 105, 607–620 (2024). 10.1002/cyto.a.24883 38943226 PMC11425855

[15] Niver A. P. Bruza P. Pogue B. W., “Optimized scintillation imaging in low dose rate and bright room light conditions,” Biomed. Phys. Eng. Express 11, 017002 (2024). 10.1088/2057-1976/ad91bb PMC12668778 39536339

[16] Wang S. et al., “Robust real-time segmentation of bio-morphological features in human Cherenkov imaging during radiotherapy via deep learning,” Med. Phys. 52(8), e18002 (2025). 10.1002/mp.18002 40781822

[17] Jia M. et al., “Time-gated single-pixel imaging of Cherenkov emission from a medical linear accelerator,” Opt. Lett. 49, 2425–2428 (2024). 10.1364/OL.518624 38691735 PMC12746906

[18] Jia M. et al., “Photon-limited Cherenkov imaging of radiation therapy dose,” Opt. Lett. 48, 1918–1921 (2023). 10.1364/OL.485668 37221799 PMC10914388

[19] Andreozzi J. M. et al., “Improving treatment geometries in total skin electron therapy: experimental investigation of linac angles and floor scatter dose contributions using Cherenkov imaging,” Med. Phys. 45, 2639–2646 (2018). 10.1002/mp.12917 29663425 PMC12964553

[20] Hunter D. I. et al., “Intermediate tissue oxygen level is required to observe murine FLASH skin sparing,” bioRxiv, preprint (2025).

[21] Cao X. et al., “Using hydrated electron temporal measurement as dosimetry of individual electron FLASH beam pulses,” Phys. Med. 142, 105725 (2026). 10.1016/j.ejmp.2026.105725 41494333 PMC12980768

[22] National Center for Interventional Biophotonic Technologies, https://ncibt.ucdavis.edu/.

[23] Schie I. W. et al., “High-throughput screening raman spectroscopy platform for label-free cellomics,” Anal. Chem. 90, 2023–2030 (2018). 10.1021/acs.analchem.7b04127 29286634

[24] Schultz C. Popp J., “Raman imaging of molecular groups in the wavenumber silent region,” Anal. Bioanal. Chem. 418, 483–497 (2026). 10.1007/s00216-025-06029-1 40796983 PMC12783204

[25] Giamarellos-Bourboulis E. J. et al., “Blood biomarker and raman analysis of leukocytes in the multicenter intelligence trials: at the intensive care unit (intelligence-1) and the emergency department (intelligence-2),” Shock 65, 15–21 (2026). 10.1097/SHK.0000000000002699 40997262 PMC12757122

[26] Asciak L. et al., “Digital twin assisted surgery, concept, opportunities, and challenges,” NPJ Digital Med. 8, 32 (2025). 10.1038/s41746-024-01413-0 PMC11736137 39815013

[27] Maier-Hein L. et al., “Surgical data science for next-generation interventions,” Nat. Biomed. Eng. 1, 691–696 (2017). 10.1038/s41551-017-0132-7 31015666

[28] Hassan M. A. et al., “Data-centric learning framework for real-time detection of aiming beam in fluorescence lifetime imaging guided surgery,” IEEE Trans. Biomed. Eng. 72, 2972–2980 (2025). IEBEAX 0018-9294 10.1109/TBME.2025.3557376 40232904 PMC13262955

[29] Alfonso-Garcia A. et al., “Label-free fluorescence lifetime imaging for real-time guidance of stereotactic biopsy: a feasibility study in brain cancer patients,” Neurooncol. Adv. 7, vdaf172 (2025). 10.1093/noajnl/vdaf172 41104039 PMC12527272

